# Emerging Therapeutic Strategies for Lung Cancer: The Role of Immunotherapy and HPV-Targeted Cancer Vaccines

**DOI:** 10.3390/vaccines13090957

**Published:** 2025-09-08

**Authors:** Krupa Bhaliya, Muneera Anwer, Ming Q. Wei

**Affiliations:** School of Pharmacy and Medical Science, Griffith University, Gold Coast, QLD 4222, Australia

**Keywords:** lung cancer, immunotherapy, cancer vaccines, HPV, checkpoint inhibitors, bacterial ghosts, tumor microenvironment, neoantigens

## Abstract

**Background/Objectives**: Lung cancer remains the leading cause of cancer-related deaths globally, with non-small-cell lung cancer (NSCLC) accounting for most cases. Although advances in targeted therapies and immunotherapy have improved outcomes, long-term survival remains limited. This review aims to explore current immunotherapeutic strategies, the evolving role of therapeutic cancer vaccines, and the emerging potential of human papillomavirus-targeted interventions in lung cancer, particularly among non-smoker populations. **Methods**: A comprehensive search of the literature was conducted using PubMed, Scopus, and Web of Science databases to identify relevant articles published between 2015 and 2024. Studies focusing on immune checkpoint inhibitors, vaccine platforms, HPV-associated lung cancer, tumor microenvironment modulation, and novel delivery systems such as bacterial ghosts were included. Relevant clinical trials and preclinical studies were critically evaluated and synthesized. **Results**: Immune checkpoint inhibitors targeting PD-1, PD-L1, and CTLA-4 have demonstrated clinical efficacy in NSCLC, yet their effectiveness is often limited by resistance mechanisms and lack of robust predictive biomarkers. Cancer vaccines, including peptide-based, mRNA, DNA, dendritic cell, and bacterial ghost platforms are emerging as complementary strategies to enhance antitumor immunity. Moreover, accumulating evidence suggests a potential association between high-risk HPV infection and lung cancer development, supporting the rationale for HPV-targeted vaccine strategies. **Conclusions**: Immunotherapy and therapeutic vaccination hold significant promise in reshaping lung cancer treatment. Advancements in vaccine design, delivery platforms like bacterial ghosts, and better understanding of HPV’s role in lung oncogenesis could support more effective, personalized immunotherapeutic approaches in the future.

## 1. Introduction

**Figure 1:**
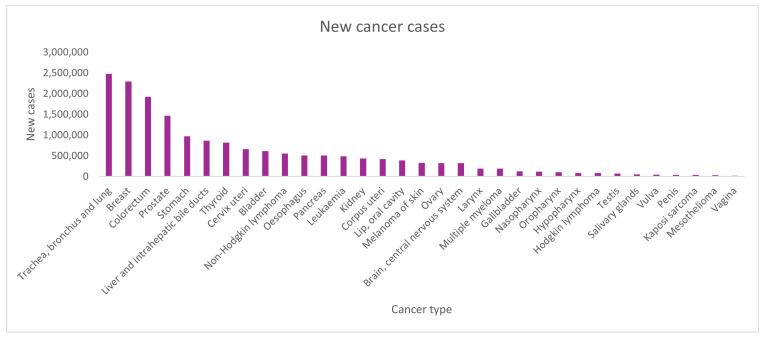
Global distribution of new cancer cases by type according to GLOBOCAN 2020. Lung cancer remains the primary burden in new cancer cases among all other cancers.

Lung cancer is the most diagnosed and deadliest cancer worldwide. According to GLOBOCAN (a cancer observatory maintained by the International Agency for Research on Cancer (IARC), which compiles and standardizes international cancer incidence, prevalence, and mortality data) 2020, there were approximately 2.2 million new lung cancer cases (11.4%) and 1.8 million deaths (18%) reported worldwide [1]. The distribution and impact of lung cancer differ greatly across regions. This variation is influenced by factors like smoking rates, environmental pollution, work-related dangers, and diet. In the United States, the lung cancer rate has steadily declined since 1990. This decrease is mainly due to effective tobacco control policies [2]. Despite these improvements, lung cancer remains a major public health issue globally. Figure 1 indicates worldwide cancer cases, where lung cancer remains among the top contributors to the global cancer burden, emphasizing the critical need for improved therapeutic strategies.

Traditional lung cancer treatments, such as surgery, radiation therapy, chemotherapy, and targeted drug therapies, have improved over time [3]. However, these treatments often face challenges like high relapse rates, the development of drug resistance, and significant side effects. Specifically, chemotherapy and radiation therapy, whether used before or after surgery, provide only modest improvements in survival and are often linked with toxic effects. As a result, the prognosis for metastatic lung cancer is still poor; five-year survival rates are around 4% [4]. Smoking causes more than 80% of lung cancer deaths, but other major risk factors include radon exposure, asbestos, long-term contact with air pollutants like polycyclic aromatic hydrocarbons (PAHs), and a family history of lung cancer [5]. The World Health Organization divides lung cancers mainly into two categories: small-cell lung cancer (SCLC), which makes up about 15% of cases, and non-small-cell lung cancer (NSCLC), accounting for approximately 80–85% of cases. NSCLC can be further divided into adenocarcinoma (LUAD), squamous-cell carcinoma (LUSC), and large-cell carcinoma (LCC), each with distinct molecular and genetic profiles. Lung cancer is a significant focus in cancer immunology research due to frequent mutations in proto-oncogenes and tumor suppressor genes, as well as issues with the immune system [3]. The limitations of conventional therapies have sparked growing interest in more personalized, immune-based treatment options. Currently, low-dose computed tomography (LDCT) is the best method for early lung cancer screening, but preventing and treating the disease remains difficult. This has led to a rise in research on immunotherapy and therapeutic cancer vaccines as promising new approaches [6]. Immunotherapy seeks to use or adjust the immune system to target tumor cells selectively, often focusing on tumor-specific antigens.

A noteworthy area of interest is the possible role of human papillomavirus (HPV) in lung cancer development, especially among non-smokers. HPV is mainly known for infecting mucosal tissues in the mouth, throat, and genital areas. HPV is primarily known for its causal role in cervical cancer, where the viral oncoproteins E6 and E7 inactivate key tumor suppressors such as p53 and Rb. Similar mechanisms are well documented in the development of anogenital and head-and-neck cancers, and these parallels suggest that HPV-driven molecular pathways may also contribute to lung carcinogenesis. Researchers suggest it may play a role in lung cancer, although its exact contribution is still being studied [7]. Other viruses linked to lung cancer include retroviruses, Epstein–Barr virus, and human immunodeficiency virus. However, HPV is notable for its presence in about 31% of lung cancer cases [8]. Advanced molecular biology techniques have shown higher HPV rates in malignant lung tissues compared to benign or normal samples. High-risk HPV strains, especially HPV16 and HPV18, along with their viral oncoproteins E6 and E7, are thought to be key in the cancer process [9]. This review aims to examine new treatment strategies for lung cancer, focusing on immunotherapy and HPV-targeted cancer vaccines. It will look closely at the shortcomings of current treatments, highlight recent advancements in immunological approaches, and evaluate evidence about HPV’s role in lung cancer development. By integrating current findings, the review seeks to identify promising targeted therapies that might improve clinical outcomes and guide future research efforts.

## 2. Epidemiology and Risk Factors of Lung Cancer

Lung cancer is the one of the most diagnosed cancers worldwide and the leading cause of cancer-related death. It accounted for about 13% of all new cancer cases in 2020 [1]. It is the most common cancer among men and the second most common among women, with around 2.2 million new cases worldwide that year. Additionally, lung cancer caused about 1.8 million deaths in 2020, highlighting its heavy impact on global public health [10]. Differences in lung cancer rates show how smoking habits and tobacco control measures vary by region. While incidence has declined in high-income countries due to reduced smoking, rates are rising in low- and middle-income regions, particularly in the Asia-Pacific, where non-smoking-related cases are increasingly reported [11]. These variations mainly result from historical tobacco-use patterns and the effectiveness of public health policies aimed at reducing smoking [12]. Research shows that strong nicotine control programs can lower lung cancer deaths by up to 40%, emphasizing the importance of ongoing tobacco control efforts [13].

Tobacco smoking has long been known as the main risk factor, accounting for over 80% of lung cancer cases each year [14]. Recent research stresses the need to consider all related risk factors without bias. Smokers face a 10- to 15-fold higher risk of developing lung cancer compared to non-smokers, with about 85% of cases linked to active smoking [15]. The risk is higher not only for current smokers but also for former smokers, people exposed to second-hand smoke, and users of other tobacco products [12]. The harmful substances in tobacco smoke cause destructive, growth-promoting, and transforming changes in bronchial cells, leading to cancer via genetic and epigenetic changes [16]. Studies have shown that these carcinogens harm various oncogenes and tumor suppressor genes, especially in lung tissue [17]. For non-smokers, second-hand smoke is the main risk factor, highlighting the need for effective smoke-free policies [18]. In addition to tobacco, air pollution has become a major environmental risk factor for lung cancer. Elderly people living in urban areas with high levels of fine particulate matter (PM2.5) face increased risk [19]. Research shows a dose–response relationship between PM2.5 exposure and lung cancer rates, with some studies finding increased risk even below current regulatory limits [20].

Occupational and environmental exposures also significantly raise lung cancer risk. For example, exposure to asbestos alone increases the risk of developing lung cancer by about 3 to 10 times, a risk that rises dramatically when combined with cigarette smoking—over a 50-fold increase in some studies [21,22]. Other workplace hazards linked to higher lung cancer risk include silica dust, arsenic, and certain industrial chemicals [23]. Beyond chemical and particulate exposures, viral infections are an emerging area of interest in lung cancer etiology. Among these, human papillomavirus (HPV) has gained attention, particularly in non-smoking populations. HPV, a DNA virus known for its role in anogenital and oropharyngeal cancers, has also been implicated in pulmonary carcinogenesis. High-risk HPV subtypes—especially HPV16 and HPV18—have been identified in malignant lung tissues, and their E6 and E7 oncoproteins are believed to disrupt p53 and Rb tumor suppressor pathways, promoting uncontrolled cell proliferation [8,9]. Multiple molecular epidemiological studies have reported HPV DNA in lung tumor samples, with prevalence estimates ranging from 20% to over 30% depending on geographic and population-specific factors [7]. A meta-analysis further supports the association between HPV and lung cancer, particularly among non-smokers and women. Moreover, the use of advanced detection techniques such as polymerase chain reaction (PCR) has improved the sensitivity of HPV identification in lung tissue samples, lending further credibility to these findings [24]. Although the causal role of HPV in lung cancer remains under investigation, the observed correlations suggest a potentially important etiological factor, especially in regions with high HPV prevalence and among patient populations without significant tobacco exposure. Recognition of HPV’s oncogenic potential in lung cancer may open new avenues for prevention, including targeted vaccination and immunotherapy strategies.

### Controversies and Limitations of Current Evidence

Although multiple studies have reported a significant association between high-risk HPV infection and lung cancer, particularly in non-smokers, it remains unclear whether HPV plays a causative role. Detection of HPV DNA or proteins in tumor samples may reflect correlation rather than direct oncogenic involvement. Confounding factors, such as geographic differences, sample contamination, and methodological variability, complicate interpretation. Furthermore, the relatively low prevalence of HPV-positive lung cancers compared to cervical and head-and-neck cancers suggests that HPV may act as a co-factor rather than a primary driver. Future studies employing standardized assays and large, multicenter cohorts are required to clarify whether HPV is causally involved in lung carcinogenesis or merely associated with disease in certain populations.

## 3. Molecular Classification and Pathogenesis

Lung cancer falls into two main categories: non-small-cell lung cancer (NSCLC) and small-cell lung cancer (SCLC). Each category has different molecular and clinical profiles. NSCLC makes up about 85% of lung cancers and has three main subtypes: lung adenocarcinoma (LUAD), lung squamous-cell carcinoma (LUSC), and large-cell carcinoma (LCC). SCLC accounts for the remaining 15%. It is known for rapid progression, high potential to spread, and neuroendocrine characteristics [25]. Lung cancer arises from diverse molecular alterations, including mutations in KRAS, EGFR, TP53, and chromosomal rearrangements such as ALK fusions. Beyond genetic drivers, viruses are established contributors to carcinogenesis, as seen with hepatitis B virus in hepatocellular carcinoma, Epstein–Barr virus in nasopharyngeal carcinoma, and human papillomavirus in cervical and anogenital cancers. Within this broader context, HPV has emerged as a causative candidate in lung cancer [26,27].

**Figure 2:**
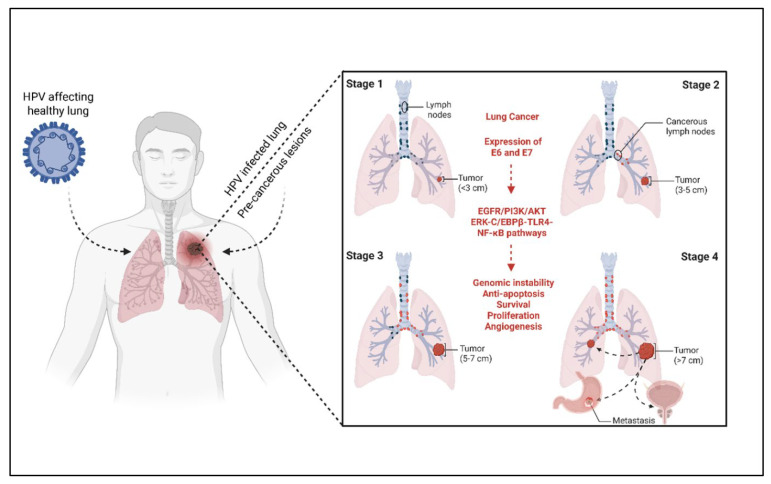
Mechanism of HPV-mediated lung cancer. The figure outlines how high-risk HPV infects lung epithelial cells and expresses E6 and E7 oncoproteins, which disrupt p53 and Rb tumor suppressor pathways. This leads to genomic instability, uncontrolled proliferation, and progression through various stages of tumor development, ultimately resulting in metastasis.

High-risk human papillomavirus (HPV) types, particularly HPV-16 and HPV-18, have been implicated in lung carcinogenesis through the expression of oncoproteins E6 and E7. These proteins inactivate key tumor suppressors such as p53 and Rb, promoting uncontrolled cell proliferation and genomic instability. Figure 2 illustrates the proposed mechanism by which HPV enters the lung epithelium, disrupts cellular regulatory pathways, and contributes to tumor progression and metastasis.

### 3.1. SCLC vs. NSCLC Subtypes (LUAD, LUSC, LCC)

LUAD usually develops in the outer regions of the lung and is the most common type among non-smokers. It is often linked to mutations in EGFR, KRAS, ALK, BRAF, and MET. LUSC is found in the center of the lung and is strongly associated with smoking; it shows changes in TP53, PIK3CA, CDKN2A, and SOX2. LCC is a diagnosis made when other types are excluded; it represents undifferentiated tumors lacking glandular or squamous features. This type has a high mutation rate but few mutations that can be targeted [28]. SCLC is marked by the loss of function in both TP53 and RB1. It often shows neuroendocrine markers like ASCL1 and NEUROD1. Molecular profiling of SCLC has led to four subtypes based on the expression of transcription factors: ASCL1-high (SCLC-A), NEUROD1-high (SCLC-N), POU2F3-high (SCLC-P), and an inflammatory subtype (SCLC-I). The inflammatory subtype may have a better response to immunotherapy [29,30].

### 3.2. Genetic Mutations and Immune Evasion Mechanisms

The mutation patterns in LUAD and LUSC greatly affect tumor biology and treatment options. Immune exclusion refers to a tumor immune evasion strategy where immune cells, particularly cytotoxic T lymphocytes, are physically prevented from infiltrating the tumor parenchyma, often due to stromal barriers, abnormal vasculature, or immunosuppressive cells within the tumor microenvironment. In LUAD, EGFR mutations are linked to immune exclusion and a lower tumor mutation burden (TMB), which leads to a reduced response to immune checkpoint inhibitors (ICIs) [31]. On the other hand, KRAS-mutant LUAD, especially with concurrent TP53 mutations, often shows high PD-L1 expression and immune cell infiltration. This can enhance responsiveness to ICIs [32]. Immune evasion is a key feature of lung cancer. Tumors reduce the expression of MHC class I molecules. They secrete immunosuppressive cytokines, like TGF-β and IL-10, and recruit regulatory T cells and myeloid-derived suppressor cells (MDSCs) to limit the activity of cytotoxic T cells [33].

## 4. Immunotherapy in Lung Cancer

**Figure 3:**
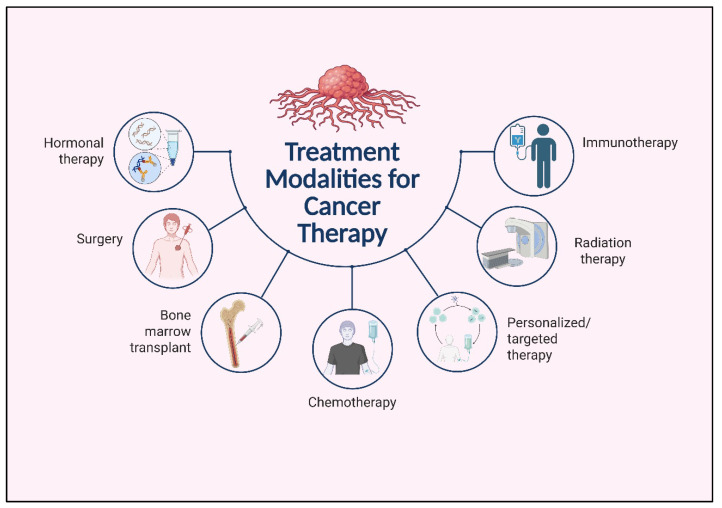
Comprehensive overview of treatment modalities for cancer.

Lung cancer treatment relies on a combination of traditional and emerging modalities, including surgery, chemotherapy, radiotherapy, targeted therapy, immunotherapy, and cancer vaccines. As shown in Figure 3, these treatment strategies vary depending on cancer stage, molecular profile, and patient-specific factors.

**Figure 4:**
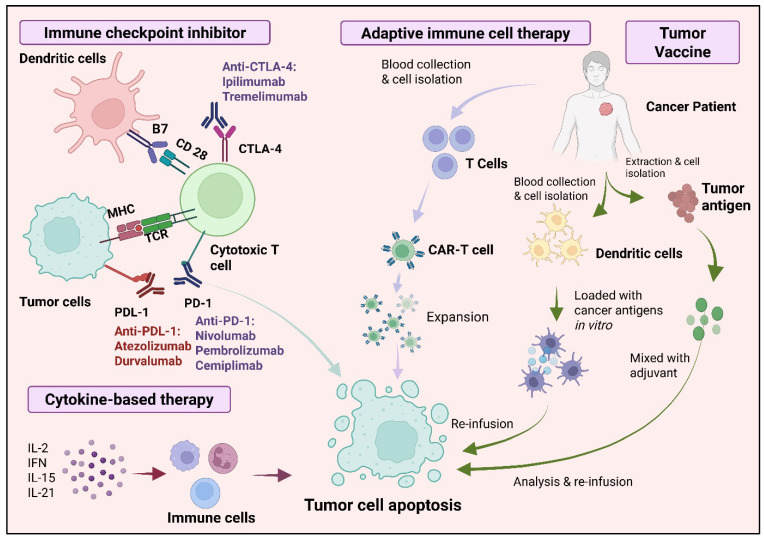
Mechanisms of immunotherapy in lung cancer. Checkpoint inhibitors, such as anti-PD-1, anti-PD-L1, and anti-CTLA-4, reactivate exhausted T cells by blocking inhibitory immune checkpoints. This enhances cytotoxic T cell function and promotes tumor cell elimination within the immunosuppressive tumor microenvironment.

Immunotherapy has greatly changed the treatment options for lung cancer, especially non-small-cell lung cancer (NSCLC). It offers better survival rates and lasting responses in certain patient groups. Among the different immunotherapy approaches, immune checkpoint inhibitors (ICIs) that target programmed cell death protein-1 (PD-1), its ligand PD-L1, cytotoxic T-lymphocyte-associated antigen-4 (CTLA-4), and the recently studied lymphocyte-activation gene 3 (LAG-3) have shown significant effectiveness [34,35]. These drugs work by blocking immune-suppressive pathways used by tumors to hide from the immune system, thus restoring T cell activity against tumors. T cell activation is carefully regulated by a complex interaction of co-stimulatory and co-inhibitory signals. Immune checkpoints help manage this balance to prevent excessive immune reactions and autoimmunity. However, in cancer, tumor cells often manipulate these checkpoints to weaken T cell function [36]. Therapeutic antibodies that block PD-1/PD-L1 and CTLA-4 pathways enable T cell growth and cytokine production, making tumors more recognizable to the immune system. Nivolumab and pembrolizumab, which target PD-1, are now approved for advanced NSCLC and have shown lasting clinical responses in certain patients [37]. Immunotherapy has emerged as a cornerstone in lung cancer treatment, particularly through immune checkpoint blockade targeting PD-1/PD-L1 and CTLA-4 pathways. These therapies restore the ability of T cells to recognize and eliminate tumor cells. Figure 4 illustrates the mechanisms by which checkpoint inhibitors modulate the tumor–immune interaction within the lung tumor microenvironment.

In addition to checkpoint blockers, other immunotherapy methods are gaining popularity. These include chimeric antigen receptor T cell (CAR-T) therapy, cytokine-induced killer (CIK) cells, and combination therapies that use different immune-boosting agents together. CAR-T cell therapy, while previously studied mainly in blood cancers, is being explored for solid tumors like lung cancer. However, challenges such as suppression in the tumor microenvironment (TME) and differences in tumor antigens remain [38]. Combining immunotherapy with other treatments, such as chemotherapy or antiangiogenic therapies, has shown promise in increasing immune responses and reducing resistance [39]. The tumor microenvironment (TME) significantly affects immune responses. Elements within the TME, such as regulatory T cells, myeloid-derived suppressor cells, and immunosuppressive cytokines, create an environment that makes effective immunotherapy difficult. Addressing these challenges is crucial for enhancing treatment responses and is a key focus of current research [40]. Despite positive results, there are still challenges. One major issue is the inconsistent predictive value of PD-L1 expression in both NSCLC and small-cell lung cancer (SCLC). While PD-L1 is often more abundant in immune cells infiltrating tumors than in the tumors themselves, it has not reliably predicted clinical benefit in SCLC, which limits its usefulness as a biomarker [41]. As a result, researchers are looking for alternative targets. Molecules like B7-H3 and CD47 are emerging as promising options. B7-H3 is believed to limit T cell entry and support tumor growth, while CD47 serves as a “don’t eat me” signal that stops the immune system from attacking tumor cells. Blocking CD47 has shown promise in improving the removal of SCLC cells by the immune system [42].

Immunotherapy is a groundbreaking approach in treating lung cancer, with ICIs being a central part of current clinical practices. Although immune checkpoint inhibitors have transformed lung cancer treatment, they are associated with immune-related adverse events (irAEs), including pneumonitis, colitis, dermatitis, and endocrinopathies. These off-target toxicities distinguish immunotherapy from chemotherapy and targeted therapies, highlighting the importance of biomarkers that can predict not only treatment response but also toxicity risk. Table 1 summarizes the challenges in immunotherapy and their proposed strategies with ongoing clinical trials. Developing new targets, optimizing combination therapies, and gaining a better understanding of immune evasion and TME will be vital for maximizing effectiveness and expanding the advantages of immunotherapy to more patients.

## 5. Cancer Vaccines in Lung Cancer

### 5.1. Traditional and Neoantigen-Based Vaccines

Cancer vaccines have traditionally targeted infectious diseases. Recently, they were being adapted to stimulate immune responses against cancer. Early therapeutic cancer vaccines focused on tumor-associated antigens (TAAs), which are self-proteins overexpressed in tumors. However, these vaccines often failed to trigger effective new T cell responses because of immune tolerance mechanisms [47]. In contrast, neoantigen-based vaccines target tumor-specific antigens (TSAs) that come from somatic mutations and are not affected by central or peripheral tolerance. They provide a strong way to trigger targeted immune responses with limited off-target effects. These vaccines also tend to avoid unwanted damage to normal tissues [48,49]. Finding and selecting appropriate neoantigens that are uniquely expressed by cancer cells is critical for vaccine effectiveness. Unlike TAAs, neoantigens come from mutated proteins that the immune system recognizes as foreign, usually leading to strong CD4⁺ and CD8⁺ T cell responses [49]. However, tumors often contain numerous mutations, which increases the chances of finding immunogenic neoantigens in cancers with high mutational burdens. Even with careful target selection, the immunosuppressive tumor microenvironment (TME) poses a significant challenge. This environment is marked by low oxygen, lack of nutrients, acidic pH, high levels of reactive oxygen species, and dense infiltration by regulatory T cells [50].

In addition to TME challenges, several other factors limit vaccine performance. Tumor fibroblasts, myeloid-derived suppressor cells, and a thick extracellular matrix hinder T cell infiltration [50]. Low levels of antigen-presenting cells (APCs), mechanisms for antigen escape, and the overall tumor mutational burden also restrict vaccine responses. Although neo-antigen strategies have made great strides, many remain in preclinical stages. Vaccination alone may not be enough without addressing the broader immunosuppressive context. It is important to combine adjuvants, proper antigen selection, and effective delivery systems to overcome these barriers. Currently, the most advanced vaccine platforms include cellular vaccines, which can be based on whole tumor cells or dendritic cells (DC), synthetic long-peptide (SLP) vaccines, and nucleic acid-based vaccines (RNA or DNA) [51]. Table 2 summarizes different vaccine platforms for lung cancer in the developmental stage.

### 5.2. Nucleic Acid Vaccines

Nucleic acid vaccines use mRNA or DNA that encode tumor antigens, which host cells take up and translate in the body. mRNA vaccines allow for quick development and strong immune response, making them ideal for personalized neoantigen strategies or general use [60]. Crucially, mRNA allows for post-translational modifications like glycosylation and phosphorylation, which are often essential for proper protein folding and antigen presentation. Directly injecting mRNA into lymph nodes has been shown to activate APCs, marked by increased CD86 and IL-12 expression, and stimulate strong CD4⁺ and CD8⁺ T cell responses [61]. DNA vaccines have advantages in terms of stability and storage. Plasmid DNA can produce multiple mRNA copies once transcribed in the nucleus. However, this requirement for entering the nucleus delays the production of antigens and lowers their immune response compared to mRNA vaccines. DNA also carries a potential, though low, risk of integrating into the genome, a risk that mRNA vaccines do not have [40]. Mechanically, DNA vaccines involve inserting plasmids encoding tumor antigens into somatic cells, leading to antigen expression and presentation through major histocompatibility complex (MHC) I and II pathways [62]. Antigens can be expressed inside cells, secreted, or released in apoptotic bodies, stimulating both humoral and cellular immunity. The best delivery method seems to be direct transfection of APCs, especially through intradermal injection [63]. DNA vaccines also activate innate immune pathways—through TLR9 signaling and STING activation—resulting in the release of chemokines, cytokines, and type I interferons [64]. These vaccines are cost-effective, stable, safe, and specific, yet their moderate immune response has limited their success in clinical settings so far [65,66].

### 5.3. Peptide-Based Cancer Vaccines

Peptide vaccines generally consist of 20–30 amino acids that represent TAAs or TSAs, including neoantigens or cancer/testis antigens. These peptides are designed to trigger both CD8⁺ and CD4⁺ T cell responses via MHC class I and II presentation [67]. Synthetic long peptides (SLPs) are preferred because their processing inside cells leads to stronger T cell activation compared to short peptides [68]. To boost immune responses, peptide vaccines are formulated with adjuvants like aluminum salts, MF59, CpG motifs, poly-ICLC, and cyclic dinucleotides. Heteroclitic peptides, modified to enhance MHC binding, further increase immune response. Because peptides can degrade quickly in the body, they are often delivered using stabilizing carriers like liposomes or PLGA nanoparticles [69]. To tackle the immunosuppressive TME, future cancer vaccine strategies should continue to optimize antigen selection, delivery methods, and adjuvant systems. By combining strong neoantigen identification with advanced vaccine design, we can enhance the chances of generating lasting, specific immune responses in lung cancer patients.

### 5.4. Bacterial Ghost-Based Cancer Vaccine

Bacterial ghosts (BGs) are empty bacterial cell envelopes generated by genetic engineering or chemical methods. They maintain the native surface antigens and structural integrity of bacteria but are devoid of cytoplasmic contents, making them a promising platform for safe and immunogenic antigen delivery. BGs can be engineered to carry tumor-associated antigens or adjuvants and have shown efficacy in preclinical models for inducing strong Th1-skewed immune responses, activating antigen-presenting cells, and promoting cytotoxic T cell activation. Given their intrinsic adjuvanticity and ability to mimic pathogen-associated molecular patterns (PAMPs), BG-based vaccines are particularly promising for lung cancer immunotherapy. While research is still emerging, recent studies have explored their application in mucosal immunization and intratumoral delivery in lung and colorectal cancer models. Many probiotic bacteria are also used for bacteria-based cancer vaccines. Their use could complement existing cancer vaccine strategies by enhancing innate and adaptive immune activation with minimal systemic toxicity [53,70].

Peptide vaccines, killed virus, and BGs primarily promote antigen-specific CD4⁺ T helper responses and CD8⁺ expansion with cytokine secretion. True cytotoxic activity requires endogenous antigen synthesis (e.g., live virus, mRNA, DNA vaccines). Therefore, BGs and peptide vaccines serve more as immune modulators than direct inducers of cytotoxic clearance. While bacterial ghosts and peptide vaccines can induce CD8⁺ T cell expansion and IFN-γ secretion, these responses may not translate into direct cytotoxic activity against tumor cells unless combined with antigen delivery strategies that support endogenous processing.

## 6. Vaccine Adjuvants for Enhancing Cancer Immunity

The effectiveness of cancer vaccines relies not just on choosing tumor-specific antigens but also on having suitable adjuvants that can enhance and guide the immune response. Adjuvants improve the immunogenicity of vaccines by stimulating antigen-presenting cells (APCs), promoting antigen uptake, and initiating both innate and adaptive immunity. This section categorizes vaccine adjuvants into traditional and emerging classes, summarizing how they work and their clinical relevance in cancer immunotherapy.

### 6.1. Traditional Adjuvants: TLR Agonists, Aluminum Salts, Poly-ICLC

Conventional adjuvants have formed the basis for many immunotherapies. Among the most used are toll-like receptor (TLR) agonists, aluminum-based compounds, and synthetic double-stranded RNA analogs like polyinosinic–polycytidylic acid stabilized with polylysine and carboxymethylcellulose (poly-ICLC). These substances mainly work by activating pattern recognition receptors (PRRs), signaling the immune system to respond to perceived threats [71]. TLR agonists, like CpG oligodeoxynucleotides (TLR9), imiquimod (TLR7), and monophosphoryl lipid A (TLR4), have shown strong immunostimulatory effects by improving dendritic cell (DC) maturation and boosting type I interferon secretion [72]. Poly-ICLC, which mimics viral RNA, works through TLR3 and the melanoma differentiation-associated protein 5 (MDA5) pathway to stimulate cytokine production and T cell activation. Aluminum salts, while traditionally used in preventive vaccines, are less effective in generating the cytotoxic T lymphocyte (CTL) responses needed for tumor rejection, but they may still support humoral immunity through inflammasome activation. While adjuvants such as alum are effective in enhancing humoral responses, their role in promoting T cell responses in tissue-resident settings remains controversial, with some formulations raising concerns of excessive inflammation or tissue adhesion, particularly in the context of HPV vaccines.

### 6.2. Emerging Adjuvants: STING Agonists, CD40, Cytokines, and Inorganic Nanoparticles

Recent studies have discovered a new generation of adjuvants that could overcome the immunosuppressive tumor microenvironment (TME) and enhance antigen presentation. These include STING agonists, CD40 agonists, immune-boosting cytokines (e.g., GM-CSF), and engineered inorganic nanoparticles.

STING (stimulator of interferon genes) agonists are a promising group of adjuvants that activate strong type I interferon responses through cytosolic DNA sensing pathways. Natural cyclic dinucleotides (CDNs) and synthetic derivatives such as cyclic GMP–AMP (cGAMP) have shown success in activating antitumor immunity through STING-mediated signaling [73,74,75]. These agents enhance DC maturation and CD8+ T cell priming and increase the infiltration of effector T cells into tumors.

CD40 agonists stimulate DCs and B cells, improving antigen processing and cross-presentation. Although they were initially studied as standalone treatments, CD40 ligands have shown better results when combined with TLR agonists in preclinical vaccine models [67]. However, their clinical use has been limited due to potential toxicity and variable pharmacokinetics. Granulocyte-macrophage colony-stimulating factor (GM-CSF) is one of the most studied cytokine adjuvants. It promotes DC recruitment and activation at the vaccination site [76]. Despite positive preclinical results, clinical trials have had mixed outcomes. GM-CSF can lead to an increase in immunosuppressive myeloid-derived suppressor cells (MDSCs) under certain conditions, which may counteract its intended effects [77,78,79]. These conflicting effects highlight the need for better dosing strategies and combination treatments.

Inorganic nanoparticles, such as titanium dioxide (TiO_2_) and manganese-based adjuvants, have emerged as innovative delivery systems and immune boosters. For example, spiky TiO_2_ nanoparticles disrupt phagolysosomal membranes during APC uptake, improving antigen cross-presentation and T cell priming [80,81]. Manganese can enhance STING signaling and cytosolic DNA sensing. Manganese-based colloidal gels (e.g., MJ) significantly improved both humoral and cellular immune responses in preclinical cancer models [82]. Other experimental platforms include polymeric nanoparticles (e.g., PC7A) that combine antigen delivery with STING activation and better T cell responses [83,84]. Additionally, compounds that modify the mevalonate pathway—like statins and bisphosphonates—are being studied for their ability to generate strong CTL responses by changing cholesterol biosynthesis and improving antigen presentation [85].

### 6.3. Clinical and Preclinical Findings

Many adjuvants have shown promise in preclinical models, but translating these results into human use remains difficult. Challenges include concerns about toxicity, differences in immune responses, and limited information on long-term safety. GM-CSF is still used in clinical trials, despite its mixed results [86]. New platforms like STING and CD40 agonists have shown potential in early studies, but they need careful optimization to prevent systemic inflammation. Similarly, controlling the physicochemical properties of nanoparticle-based adjuvants is essential to balance effectiveness with compatibility. In the end, the ideal cancer vaccine adjuvant should safely and effectively boost tumor-specific immunity, help with antigen presentation, and modulate the tumor microenvironment without causing significant off-target effects. Thoughtful design, combination strategies, and personalized approaches based on biomarkers will be crucial for the next generation of adjuvants for lung cancer vaccines.

## 7. Imidazoquinolines (IMDs) as Cancer Immunomodulators

### 7.1. Mechanisms: TLR7/8 Activation

Imidazoquinolines (IMDs) are a class of synthetic small-molecule agonists that activate toll-like receptors 7 and 8 (TLR7/8), which are localized in endosomal compartments of innate immune cells. Upon activation, these receptors trigger a MyD88-dependent signaling cascade involving IRAK1/4 and TRAF6, culminating in the activation of NF-κB and mitogen-activated protein kinases (MAPKs), as well as interferon regulatory factors (IRFs) [87,88]. This cascade stimulates the production of proinflammatory cytokines such as IL-12 and type I interferons (IFNs), particularly IFN-α, which enhance antigen presentation, promote dendritic cell (DC) maturation, and improve cytotoxic T lymphocyte (CTL) activation [89]. These mechanisms have established IMDs as potent immunomodulators in oncology. However, the biological effects of TLR7/8 ligands can vary depending on tumor type and microenvironment. For instance, while some preclinical studies demonstrated tumoricidal activity through enhanced adaptive immunity, others have shown that TLR7/8 activation might paradoxically promote lung tumor progression in certain contexts [90,91]. These dichotomous findings underscore the necessity of precise targeting strategies and context-aware therapeutic design [92,93].

### 7.2. FDA-Approved Agents: Imiquimod and Resiquimod

The first FDA-approved IMD was imiquimod (R837), licensed in 1997 for the topical treatment of genital and perianal warts, actinic keratosis, and later, superficial basal-cell carcinoma [94]. It selectively activates TLR7, inducing robust local immune responses through type I IFN production. Building upon this success, resiquimod (R848) was developed as a more potent TLR7/8 dual agonist—estimated to be over 100 times more effective in TLR activation than R837 [95]. While R848 has not yet received FDA approval, it has shown substantial immunostimulatory capacity in preclinical models and early-phase clinical trials. Systemic administration of R848 in patients with metastatic solid tumors (e.g., colorectal, breast, ovarian, and cervical cancers) led to disease stabilization in a subset of cases and elevated serum levels of IFN-γ and TNF-α, indicative of heightened immune activation [96]. However, systemic cytokine release also induced significant adverse effects, limiting further clinical progression. These challenges have redirected focus toward topical or targeted delivery methods for safer, localized immunomodulation [95].

### 7.3. Applications as Vaccine Adjuvants and Topical Immunotherapies

IMDs such as imiquimod and resiquimod are increasingly used as vaccine adjuvants, particularly in therapeutic cancer vaccines. More than 100 clinical trials have explored their role as immune potentiators in vaccines for cancer (e.g., dendritic cell-based immunizations), infectious diseases (e.g., hepatitis B), and respiratory illnesses (e.g., influenza) [97]. Recent studies suggest that combining IMDs with tumor-associated or viral antigens—such as HPV16 E7—may offer a viable immunotherapeutic strategy for HPV-associated lung cancers. Topical IMD formulations are of particular interest due to their ability to generate robust local inflammation and antigen-specific immune responses without systemic toxicity. These benefits are driven by TLR7/8-mediated cytokine release, particularly IL-12 and type I IFNs, which activate plasmacytoid DCs, conventional DCs, macrophages, and monocytes [95,98]. These mechanisms support their dual utility in both topical immunotherapy and vaccine adjuvancy.

### 7.4. Future Directions and Clinical Integration

Despite their promising effects on the immune system, the use of IMDs in the whole body is still limited due to the risk of cytokine storms and other inflammatory reactions. Future strategies focus on localized delivery systems, like nanoformulations or injections directly into tumors, to improve effectiveness while reducing side effects [96]. Table 3 illustrates the mechanism of action and clinical status of traditional and emerging adjuvants. Research is also looking into changing the structure of IMDs to adjust TLR specificity and improve signaling strength [95].

## 8. Future Perspectives and Research Directions

### 8.1. Personalized Neoantigen Vaccines and Immunotherapy

Personalized cancer immunotherapy, especially through neoantigen-based vaccination strategies, has quickly moved from concept to early clinical practice. These methods, particularly when combined with immune checkpoint inhibitors, are creating new possibilities for cancer treatment. As this area develops, future research will likely focus on refining and broadening four key areas: personalized vaccine strategies, combinations of different immunotherapies, integrating virology with oncology, and developing predictive biomarkers to guide treatment. Table 4 represents the feasibility and timeline of immunotherapeutic strategies. 

Personalized cancer vaccines are designed to take advantage of tumor diversity by targeting patient-specific neoantigens that arise from non-synonymous mutations [99]. Unlike shared tumor-associated antigens, neoantigens are not found in healthy tissues. This significantly lowers the risk of central immune tolerance and autoimmune reactions. Several types of vaccine platforms have been tested, including synthetic long peptides, mRNA, DNA, dendritic cell-based vaccines, and viral vector vaccines. Currently, mRNA platforms lead due to their flexibility, rapid production, and safety [100].

Clinical data show that neoantigen vaccines are feasible and can provoke immune responses across multiple tumor types. For example, the KEYNOTE-942 trial showed that the mRNA-4157 vaccine from Moderna and Merck, used alongside pembrolizumab, led to a 44% decrease in melanoma recurrence compared to checkpoint blockade used alone [101]. Similarly, trials in glioblastoma and non-small-cell lung cancer have found that personalized peptide vaccines can generate lasting CD^4+^ and CD^8+^ T cell responses when combined with standard treatments [102].

Future work on vaccine strategies will involve improving neoantigen prediction algorithms by using multiomics data and machine learning. Additionally, researchers will optimize delivery methods, such as lipid nanoparticles and viral vectors, while working to lower the time and cost of making individualized vaccines. Progress in RNA technology, including self-amplifying mRNA and circular RNA platforms, could enhance antigen expression and reduce dosage needs, which may improve effectiveness and accessibility [103].

### 8.2. Multi-Modal Immunotherapy Combinations

A promising future direction is combining neoantigen vaccines with other immunotherapies. Checkpoint inhibitors, like anti-PD-1 and anti-CTLA-4 antibodies, help T cell function by preventing exhaustion. When used alongside personalized vaccines, these agents show synergistic effects. Vaccines expand the antigen-specific T cell pool while checkpoint inhibitors sustain their activity [104].

In addition to checkpoint inhibitors, combining neoantigen vaccines with adoptive T cell therapies, such as chimeric antigen receptor (CAR) T cells or tumor-infiltrating lymphocytes (TILs), is gaining interest. Vaccine priming might improve the efficacy of infused T cells by broadening the pool of relevant antigen-specific clones. Oncolytic viruses, which specifically target tumor cells, can increase local inflammation and antigen presentation, acting as additional vaccine boosters [105].

Timing will be crucial for the success of these combinations. Research suggests that administering vaccines before or at the same time as checkpoint inhibitors may maximize effectiveness. A delayed administration of checkpoint inhibitors could reduce their synergistic effect due to early T cell exhaustion [106]. Ongoing clinical trials are exploring the best schedules and dosing for these combinations.

### 8.3. Integrating Virology and Oncology

Another emerging focus is to combine virology with personalized immunotherapy. Virus-related cancers, such as cervical cancer (HPV), nasopharyngeal carcinoma (EBV), and Merkel cell carcinoma (polyomavirus), provide additional targets through viral oncoproteins. These viral antigens make ideal immunologic targets, since they are foreign and highly conserved, allowing for both personalized and semi-universal vaccine designs [107].

Moreover, researchers are considering viral vectors as delivery vehicles for neoantigen vaccines. Their strong ability to provoke immune responses and infect antigen-presenting cells is beneficial [105]. Modified adenoviruses, poxviruses, and lentiviruses are being tested in both lab and clinical settings. Oncolytic viruses may offer two benefits: they can directly destroy tumors and stimulate the release of immunogenic antigens, enhancing the effectiveness of personalized vaccines when used together. A future strategy might involve engineering synthetic viruses that carry both neoantigens and immunomodulatory genes like GM-CSF or IL-12. This vitro-immunotherapeutic approach aims to boost both innate and adaptive immune responses while reducing systemic toxicity [100].

### 8.4. Predictive Biomarkers and Precision Approaches

A vital future focus is identifying and validating biomarkers that predict which patients will benefit the most from neoantigen vaccine therapies. Tumor mutational burden (TMB) has often served as a measure of neoantigen load, but it varies across different tumor types and individual patients [106]. Not all mutations create immunogenic peptides, and some tumors with high TMB still escape immune detection due to faulty antigen processing.

Other potential biomarkers include the clonality of neoantigens, MHC-binding affinity scores, CD^8+^ T cell density in tumors, PD-L1 expression, and IFN-γ gene signatures [108]. Circulating tumor DNA (ctDNA) is increasingly used to track treatment responses in real time. For instance, drops in ctDNA levels after vaccination are linked to better progression-free survival in melanoma and glioblastoma [100].

Advances in spatial transcriptomics and multiplexed immunohistochemistry allow researchers to map immune cells and neo-antigen expression within the tumor microenvironment. These technologies can help customize vaccine design by selecting antigens from regions rich in immune cells while steering clear of areas lacking immune activity [105].

While cancer vaccines have focused largely on host-derived neoantigens and tumor-associated antigens, HPV represents a distinct viral target in a subset of lung cancers. HPV-targeted vaccines remain largely unexplored for lung cancer, but preclinical studies suggest that vaccines targeting HPV E6 and E7 oncoproteins may enhance antitumor immunity in HPV-positive lung tumors. Translation to the clinic is limited by the relatively low prevalence of HPV-positive lung cancers and the lack of standardized diagnostic assays. Nevertheless, lessons from successful HPV-targeted vaccines in cervical and head-and-neck cancers provide a foundation for developing lung cancer vaccines aimed at viral causes. Future strategies may involve combination regimens that integrate HPV vaccines with checkpoint inhibitors or novel adjuvants to increase efficacy.

Ultimately, the aim is to create integrated biomarker models combining genomic, proteomic, and immunologic data using machine learning algorithms. These models will assist in choosing patients, formulating vaccines, and planning combination strategies. They will enable real-time adjustments to immunotherapy regimens based on changes in tumors, creating a truly dynamic precision oncology environment.

The future of cancer immunotherapy is in merging personalized neoantigen vaccines with multi-modal strategies, viral immunology, and precise biomarker frameworks. As clinical experiences increase and technology improves, we expect better access, enhanced outcomes, and adaptive treatment models to emerge. By focusing on refining antigen prediction, validating combination therapies, and expanding biomarker-guided strategies, the field is set to provide effective immunotherapy to more patients.

## 9. Conclusions

Lung cancer continues to be one of the most challenging malignancies to treat, largely due to its late-stage diagnosis, molecular heterogeneity, and complex immune evasion mechanisms. The advent of immunotherapy, particularly immune checkpoint inhibitors, has revolutionized the therapeutic landscape and significantly improved outcomes for a subset of patients. However, treatment resistance, limited biomarker precision, and variability in response highlight the need for more refined and innovative approaches.

Therapeutic cancer vaccines represent a promising adjunct to existing immunotherapies, aiming to generate durable and antigen-specific immune responses. Among these, platforms such as mRNA, DNA, dendritic cell, and bacterial ghost-based vaccines are under active investigation, each offering unique immunogenic and logistical advantages. Notably, emerging evidence implicates high-risk HPV in the pathogenesis of lung cancer, particularly among non-smoking individuals, suggesting a potential new avenue for HPV-targeted therapeutic strategies.

Future research must focus on optimizing vaccine delivery, identifying reliable predictive biomarkers, and integrating immunotherapy with conventional and targeted treatments. Platforms like bacterial ghosts also present an exciting frontier, offering both antigen presentation and innate immune activation in a single system. While BGs represent a novel avenue for vaccine delivery and immunomodulation, their safety and efficacy should be validated in preclinical and clinical settings. Moreover, reliable biomarkers are essential for predicting response and toxicity to immunotherapies, enabling more precise patient selection in biomarker discovery. Overall, the integration of immunotherapy, vaccine science, and virology holds the key to more personalized, effective, and long-lasting treatments for lung cancer. Continued exploration and clinical translation of these strategies are essential to advancing the next generation of lung cancer therapeutics.

## Figures and Tables

**Table 1:** Challenges in immunotherapy and solutions along with ongoing clinical trials.

Challenge	Description	Proposed Solutions/Strategies	Ongoing Trials	References
Lack of predictive biomarkers	PD-L1 and TMB are inconsistent predictors of ICI response	Composite biomarkers: gene expression, peripheral immune profiling	NCT03606967: multiomic biomarker analysis in NSCLC	[43]
Primary resistance	“Cold” tumors lack immune infiltration	Combination with chemo, radiotherapy, or STING agonists to enhance immunogenicity	NCT03892525: STING agonist + PD-1 blockade in solid tumors	[44]
Acquired resistance	Tumor escapes after initial ICI response	Dual checkpoint blockade (e.g., PD-1 + LAG-3), epigenetic therapy	NCT03686202: anti–PD-1 + LAG-3 in resistant NSCLC	[45]
Immune-related adverse events	Autoimmunity affecting skin, GI, lungs, etc.	IL-6 blockades, corticosteroids, better screening algorithms	NCT04167137: tocilizumab for irAEs	[46]

**Table 2:** Cancer vaccines platforms in early developmental stage.

Development Stage	Vaccine Type	Mechanism of Action	Examples/Status	References
Preclinical	Bacterial ghost vaccines	Empty bacterial envelopes deliver tumor antigens, enhance innate immunity	Animal models show promise in lung cancer	[52,53]
	Oncolytic virus-based vaccines	Selectively replicate in tumors, trigger immune response	Adenovirus and HSV-based vaccines under development	[54]
Early-phase clinical trials	mRNA vaccines	Encode neoantigens, induce antigen-specific T cell response	NCT04526899: personalized mRNA vaccines in NSCLC	[55]
	DNA vaccines	Plasmid DNA delivered by electroporation induces immune response	NCT03893955: DNA vaccine targeting surviving	[56]
	Peptide-based vaccines	Tumor-associated antigens presented on MHC molecules	NCT04397900: multi-epitope vaccine trials	[57]
Advanced/approved	Dendritic cell (DC) vaccines	Autologous DCs loaded with tumor antigens and re-infused	NCT01948141: DC vaccine with chemo in NSCLC	[58]
	Allogeneic whole-cell vaccines	Genetically modified tumor cells as broad antigen source	Belagenpumatucel-L in Phase III trials (not FDA-approved)	[59]

**Table 3:** Mechanism of action and clinical status of traditional and emerging adjuvants.

Category	Adjuvant/Agent	Mechanism of Action	Clinical Status/Notes	References
Traditional adjuvants	TLR agonists (CpG ODN, imiquimod, MPL)	Activate pattern recognition receptors (TLR7, TLR9, TLR4), enhance dendritic cell maturation, type I IFN secretion	Widely used in cancer vaccines; imiquimod FDA-approved for topical use; effective in boosting immune response	[71,72]
	Aluminum salts	Promote inflammasome activation, support humoral immunity	Common in preventive vaccines; less effective in eliciting cytotoxic T cell responses	[71]
	Poly-ICLC	Synthetic dsRNA analog activates TLR3 and MDA5 pathways, stimulates cytokine production	Used in early-stage cancer vaccine trials; boosts T cell activation	[71]
Emerging adjuvants	STING agonists (cGAMP, CDNs)	Stimulate cytosolic DNA sensing pathways, induce strong type I IFN response	Early-phase clinical trials; potent activators of antitumor immunity but require safety optimization	[73,74]
	CD40 agonists	Activate dendritic cells and B cells, enhance antigen cross-presentation	Promising in combination therapies; clinical use limited by toxicity concerns	[67]
	Cytokines (GM-CSF)	Recruit and activate dendritic cells at vaccination sites	Mixed clinical results; potential to expand immunosuppressive cells under some conditions	[76,77]
	Inorganic nanoparticles (TiO_2_, Mn-based)	Enhance antigen cross-presentation, modulate STING pathway	Preclinical stage; promising delivery platforms with immunomodulatory effects	[80,82]
Imidazoquinolines (IMDs)	Imiquimod (R837), resiquimod (R848)	TLR7/8 agonists; induce NF-κB and IRF activation, promote IL-12 and type I IFN production	Imiquimod FDA-approved for topical cancers; resiquimod in clinical trials; limited by systemic toxicity	[95,96]

**Table 4:** Clinical feasibility and timeline of immunotherapeutic strategies.

Immunotherapy Strategy	Current Stage	Estimated Clinical Timeline	Notes
Personalized neoantigen vaccines	Early clinical trials	3–7 years	mRNA and peptide vaccines showing promising immune responses and manageable safety profiles.
Combination with immune checkpoint inhibitors	Clinical trials/ongoing use	3–5 years	Synergistic effects are well documented, rapidly moving toward broader clinical adoption.
Viral vector and oncolytic virus platforms	Preclinical/early trials	5–10 years	High potential but requires optimization for safety and efficacy.
STING and CD40 agonists as adjuvants	Preclinical/Phase I trials	5–10+ years	Promising immune activation, but toxicity and delivery need refinement.
Bacterial ghost and nanoparticle-based vaccines	Preclinical	7–15 years	Innovative delivery systems with encouraging results in animal models; translation pending.
Predictive biomarker development	Early clinical/exploratory	3–7 years	Biomarkers for patient stratification improving vaccine personalization and response tracking.

## Data Availability

No new data were created or analyzed in this study.
